# Gender representation in editorial boards of international general surgery journals

**DOI:** 10.1093/bjsopen/zraa064

**Published:** 2021-04-09

**Authors:** E Gallivan, S Arshad, H Skinner, J R Burke, A L Young

**Affiliations:** School of Medicine, University of Leeds, Leeds, UK; School of Medicine, University of Leeds, Leeds, UK; St James’ University Hospital, Leeds, UK; St James’ University Hospital, Leeds, UK; Leeds Institute of Biomedical and Clinical Sciences, St James’s University Hospital, Leeds, UK; Department of Pancreatic Surgery, St James’s University Hospital, Leeds, UK

## Abstract

**Background:**

Despite women constituting over half of new doctors, gender disparity remains an issue. Surgery has shown particularly slow progress towards gender parity. This study aimed to quantify gender representation within editorial boards of the highest ranking international general surgery journals.

**Methods:**

Surgical journals were collated using two indices: SCImago Journal Rank (SJR) and Journal Impact Factor (JIF). Non-general surgery journals were excluded. Journals were contacted, requesting gender editorial team demographics. Editorial board data were collected via journal websites on 28 November 2019.

**Results:**

The top 25 general surgery journals according to SJR and JIF ranking methods were determined, identifying 28 unique journals. Editorial board data were publicly available for 27 of these 28 surgical journals, and were examined. Women accounted for 20.2 per cent (568 of 2816) of total editorial board positions. Women constituted 11 per cent (4 of 36) of editor-in-chief positions, 32 per cent (29 of 92) of deputy editors, and 19.1 per cent (369 of 1935) of general editorial board positions.

**Conclusion:**

The findings demonstrate gender disparity within editorial boards of the most prominent general surgery journals.

## Teaser

Gender disparity exists with the editorial boards of the most renowned general surgery journals. Women account for 20% of editorial board members and 11% of Editor in chief positions. The implications of such significant gender disparity are wide reaching.

Action needed to rectify disparity.

## Introduction

The number of women choosing to pursue careers in medicine continues to rise; in 2019 there were 20 per cent more UK female doctors than in 2012^1^ and 54 per cent of UK graduates joining the register were women^1^. The Association of American Medical Colleges (AAMC) reported a downwards trend of female representation with increasing job seniority in academic medicine; although 48 per cent of US medical school graduates are women, only 18 per cent of department chairs and 18 per cent of deans are women^2^. Despite women now constituting around 48 per cent of all doctors in both the UK and the USA^1^^,^^2^, they remain underrepresented in both countries in academic medicine and leadership positions^2–4^.

Although female representation in surgery is increasing gradually, in 2017 women constituted only 27 per cent of the UK surgical workforce as a whole and 32 per cent of trainees^5^^,^^6^. Similarly, the AAMC reported that, in 2017, 20.6 per cent of active US general surgeons were women^7^. There is little evidence that women are attaining senior positions within surgical academia^4^^,^^8^^,^^9^, and they remain underrepresented in wider surgical leadership positions^3^.

Women tend to progress slower in their careers and are less likely to be trained in elite research groups^10^. The Journal Impact Factor (JIF), an indicator of journal quality, has been found to correlate negatively with female authorship, suggesting that more renowned journals are less likely to publish female authors^10^. Many factors contribute to this; for example, men are invited to submit papers directly to journals at twice the rate of women^10^. It has been predicted that this gender gap could persist for centuries if no concerted action is made, particularly in the fields of surgery, computer science, physics and mathematics^10^.

Identifying and resolving barriers to female participation in surgical research and subsequent publication are important issues that need resolution^11^. To gain an understanding of the extent to which gender disparity exists in surgical research and publication, this study aimed to determine gender representation in editorial boards in top-ranking international general surgery journals.

## Methods

Two journal-ranking methods were used to determine the highest ranking international general surgery journals, JIF and SCImago Journal Rank (SJR), as used previously^12^. Journal ranking data for the year 2018 were retrieved on 9 November 2019.

JIF data were retrieved from the 2018 InCites Journal Citation Report (Clarivate Analytics, London, UK), and SJR data were retrieved via the SCImago Institutions ranking website. As no list specific to general surgery was available on either website, all surgical journals and their rankings were downloaded; those not reflecting general surgery themes were mutually agreed by all authors and removed to determine the top 25 general surgery journals according to SJR and JIF. Inclusion and exclusion criteria for determining general surgery journals are shown in *Table 1*.

**Table 1:** Inclusion and exclusion criteria for general surgery journals

Inclusion criteria	Exclusion criteria
Vascular surgery	Neurosurgery
Transplant surgery	Cardiothoracic surgery
Breast surgery	Paediatric surgery
Colorectal surgery	Orthopaedic surgery
Pancreatobiliary surgery	Obstetrics/gynaecology
Upper gastrointestinal surgery	Arthroscopy
	Plastic surgery

An internet search for editorial board information for each of the journals in the top-25 lists was conducted on 28 November 2019. The gender of staff occupying different job roles was recorded as male, female or unknown. Gender allocation was based on staff profiles. Staff recorded as unknown were excluded so as not to contribute to the total. Journal data were anonymized for the purpose of this study. Job titles were grouped into categories according to the role; job description was considered in the context of each individual editorial board (*Table 2*).

**Table 2:** Job roles detailed in online editorial board information, grouped into job role categories

Job role category	Roles included
Editors-in-chief	Editor (where this is clearly listed as the main editor)
	Editor-in-chief
Deputy/executive positions	Co-editor
	Deputy editor-in-chief
	Deputy editor
	Executive editor
	Managing editor
Senior/specialized editorial board positions	Assistant editor
	Editorial assistant
	Assistant deputy managing editor
	Section editors
	Specialty editors
	Science editor
	Corresponding editor
	Language editor
	Medical journal editor
	Surgical innovation editor
	Clinical education editor
	Associate editors
	Assisting managing editor
	Senior members
	Editorial fellows
	Research editor
	Advisory editors
	Statistical editor
	Statisticians
	Review editor
	Manuscript editor
	Senior manuscript editor
	Junior editors
Wider/general editorial board positions	Editorial board
	International editorial board
	Editors advisory council
Non-academic editorial positions	Visual abstract creative director
	Social media editor
	Creative director
	Assistant creative director
	Representatives
	Translators
External academic editorial positions	Statistical consultants
	Statistics advisor
	Consultants
	Consulting editors
	External board members
	Freelance staff
Honorary/founding positions	Honorary editors
	Editors emeriti
	Founders
	Past editors
Administrative positions	Editorial office
	Media relations staff
	Media relations manager
	Secretary
	Budget and administration staff

The editorial boards of the general surgery journals included in the top-25 lists were contacted via e-mail to request gender representation data and information regarding any organizational policies or initiatives to encourage female participation. Editorial boards were contacted in March–April 2020 using contact e-mails provided in online editorial board information. When no reply was received, a further e-mail was sent. Editors-in-chief (EICs) were also contacted where contact details were available.

## Results

The full list of included and excluded journals from the JIF and SJR lists can be found in *Tables S1* and *S2*. The 25 highest-ranking journals according to each bibliometric are listed in *Table S3*. The SJR and JIF top-25 lists were collated, identifying 28 unique general surgery journals.

### Web collection of editorial board data

Of the 28 general surgery journals in the final selection, 27 had publicly available online editorial board information. Fifty-four unique job titles were identified and categorized into eight categories of job role type (*Table 2*). Some 2942 editorial board roles were recorded as male, female or unknown. In all, 126 (4.3 per cent) were recorded as unknown and therefore excluded from the results. An overview of the gender representation data per anonymized journal can be found in *Table 3*, with details in *Table S4*. Key findings from the gender representation results are summarized per job role category below.

**Table 3:** Gender representation as deduced from web-collected data compared with data provided by editorial boards via e-mail correspondence

Role	Web collection data only (%)	**Data provided by editorial boards incorporated (%)**
Editors-in-chief	4 of 36 (11)	11.1
Deputy/executive positions	29 of 92 (32)	32.2
Senior/specialized editorial board positions	87 of 414 (21.0)	23.0
Wider/general editorial board positions	369 of 1935 (19.1)	19.8
Non-academic editorial positions	25 of 62 (40)	46.0
External academic editorial positions	41 of 173 (23.7)	24.8
Honorary/founding positions	4 of 91 (4)	3.9
Administrative positions	9 of 13 (69)	69.6
All	568 of 2816 (20.2)	21.2

Gender disparity exists within the editorial boards of the most renowned general surgery journals. Women account for 20.2 per cent of editorial board members and 11 of editor-in-chief positions. The implications of such significant gender disparity are wide-reaching.

#### Editors-in-chief

Of the 27 journals, four (15 per cent) had female EICs, with one of these journals having two EICs, (1 male, 1 female). Across the journals, of 36 EICs, four (11 per cent) were women.

#### Deputy/executive editorial board positions

Roles secondary to EIC, such as deputy editor, executive editor or deputy EIC, were listed in 22 of the 27 editorial boards. Some 32 per cent (29 of 92) of these senior roles were occupied by women.

#### Senior/specialized editorial board positions

Some 24 of the 27 editorial boards included roles categorized as senior/specialized editorial board positions. Women were present in such roles in 22 of these 24 journals. Overall, 21 per cent (87 of 414) of these senior roles were occupied by women.

#### General editorial board positions

This category included positions that were non-specific, listed as editorial board without a qualifying term. Median female representation within the general editorial boards of the journals was 16 (i.q.r. 11.1–23.5) per cent, and women constituted 19 per cent (369 of 1935) of these positions. Nine of the 27 journals had less than 10 per cent female representation in this category, although there was wide variation ranging between 0 and 43 per cent.

#### Non-academic editorial board positions

Eleven of the 27 journals had non-academic roles such as creative director and social media editor listed as part of the editorial board. Four of these journals had no women occupying these positions. Three journals scored 100 per cent female; however, they each only had one job role under this category. Some 40 per cent (25 of 62) of all non-academic editorial board roles were occupied by women.

#### External academic editorial board positions

Ten of the 27 journals included external academic positions as part of their editorial board, such as freelance staff and consultants. Overall, 24 per cent (41 of 173) of these roles were occupied by women. Some 11 per cent (19 of 173) of the external academic roles were occupied by women in a single journal.

#### Honorary/founding positions

Nineteen of the 27 journals listed honorary/founding editors such as ‘editors emeriti’. Four of these journals had female founders. Women occupied 4 per cent (4 of 91) of these roles. It was unclear whether these were active editorial board members or not, but as they were included in an editorial position online, they were included for the purpose of this analysis.

#### Administrative positions

Administrative positions were listed in 6 of the 27 journal editorial boards; 69 per cent (9 of 13) of these positions were occupied by women.

#### Total editorial board staff

The median female representation in the 27 editorial boards was 17.4 (i.q.r. 12.0–23.5) per cent. The two journals with the highest proportion of women in their editorial board staff had 51 per cent (59 of 116) and 47 per cent (30 of 64), although the latter editorial board had 10 staff members excluded from the total as they were recorded as unknown. Across the 27 journal editorial boards, 20 per cent (568 of 2816) of editorial board staff were female.

#### Relationship of female editors-in-chief with editorial board composition

The two editorial boards in this study with the highest proportion of women (journal 12 (51 per cent) and journal 4 (56 per cent)) both had female EICs. The other two journal editorial boards with female EICs also had the fourth and eighth highest proportion of women of the 27 journals.

#### Correspondence with editorial boards

All 28 general surgery journals were contacted using online editorial board contact information. Of these,19 (68 per cent) responded, two declined to provide data, and one stated they did not keep a record of the data requested. Six journals referred to their websites, confirming that online editorial board information was up to date. Three journals provided inadequate information and did not reply to follow-up e-mails. Only seven journals completed the data table provided, detailing gender representation data for each of the job role categories (*Table S5*). In summary, 13 (46 per cent) of the 28 editorial boards, when contacted, provided complete data.

**Fig. 1:**
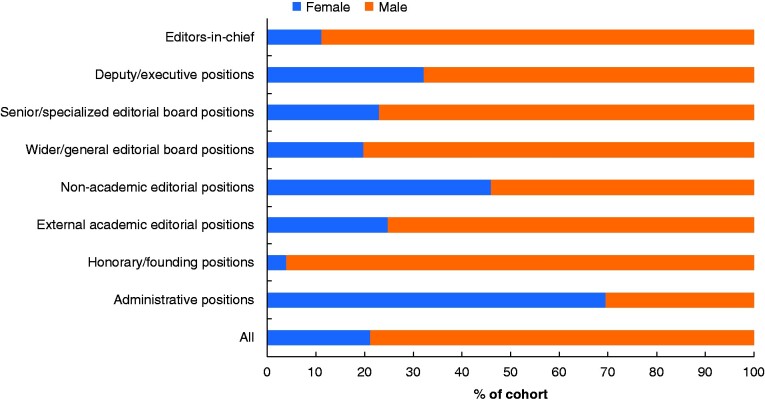
Gender split of editorial board staff members, divided by job role category, for 27 highest ranking international general surgery journals

When the data provided by editorial boards were incorporated into the existing gender representation data set by replacing the web-collected data, six of eight job categories had a positive adjustment, ranging from +0.4 +5.7 per cent, with the largest adjustment in non-academic editorial positions (*Fig. 1*). Female representation in all staff members was adjusted from 20.2 to 21.2 per cent.

## Discussion

This study has demonstrated that women continue to be underrepresented in the editorial boards of the top-ranking international general surgery journals, with 20.2 per cent of all editorial board staff recorded as female. There was only one job category where women represented the majority—‘administrative positions’—although the sample size here was small.

The significance of gender disparity should not be underestimated. Underrepresentation of women in editorial boards could reflect a lack of equal opportunities, potentially impairing peer recognition and academic advancement^12^. In the UK, the 2019 General Medical Council workforce report emphasized the importance that ‘leadership (that) fosters workplace cultures, which support our increasingly diverse workforce is also of paramount importance to meet the retention challenge’^1^. Although over half of medical students and doctors are now women, female representation in surgery has been slower to progress^9^. Gender disparity in the top surgical journal editorial boards may exacerbate publication bias, as well as impacting on career progression.

Less than 15 per cent of EIC roles were held by women. Barriers preventing women from reaching senior positions require examination. As the proportion of senior positions in editorial boards occupied by women is similar to the proportion of consultant female surgeons, this might suggest that women are reasonably represented^13^. With so few women in senior editorial positions, however, there are few role models to encourage female trainees, so a higher percentage may be necessary to promote female participation. The number of women entering surgical training is increasing, but it is unclear how this will affect female representation in senior roles.

Between 1997 and 2017 the proportion of women in editorial boards of high-impact general surgery journals increased from 5 to 19 per cent^14^. These findings, combined with the present data of 21.2 per cent female representation in 2019, show a small improvement in women occupying editorial board positions. The proportion of women on editorial boards might at least reflect the overall proportion of women in general surgery, which in 2018 was 27 per cent in the UK^5^, or even gender parity at 50 per cent. In the absence of evidence that the performance of women is inferior to that of men in these roles, moves to promote women as editorial board members should be encouraged.

Female role models have been shown to increase the likelihood of women choosing to pursue a career in surgery, and senior roles provide visible role models for aspiring female trainees^15^. The two editorial boards with the highest proportion of female editorial board members both had female EICs, suggesting that having women in this leadership role might be a factor in encouraging women to join an editorial board.

Gender disparity in these senior roles is a complex issue. It was disappointing that more than one-third of journals contacted did not provide data (9 non-responders, 1 that kept no record of staff ‘identifiable qualities, such as age, gender, political affiliation’). This study suggests that monitoring staff demographics might be a reasonable step in highlighting diversity and representation bias within editorial boards.

More information is needed about the issues that have resulted in so few women in these roles. Is it the case that women simply are not applying and, if that is true, why is this? Greater transparency by journals regarding the selection process would help.

The present study has limitations. The main limitation is the cross-sectional nature of the analysis representing a single time point for each journal, with no evaluation of changes in editorial board composition with time. Web-collected data also relied on the journal websites being accurate and updated regularly, although attempts were made to mitigate this by contacting journals. When replies were received, these were concordant with the website data.

In assigning male, female or unknown to editorial board staff members, assumptions were made using staff profiles and the use of pronouns. This method leaves the potential for error by assuming gender, and may not account for those who do not adhere to traditional pronouns or one particular gender. Where gender could not be determined, the member was recorded as unknown and later excluded from analysis. In an attempt to counteract these limitations and ensure the most accurate gender split for each editorial board was obtained, all editorial boards were contacted directly.

This study has highlighted underrepresentation of women in editorial boards of internationally renowned general surgery journals. The underlying reasons for this observation remain unclear and relatively unexplored. A fair and transparent system to enable female representation on editorial boards is needed.

## Funding

J.R.B. is in receipt of funding from the Royal College of Surgeons of England and Bowel Cancer UK. The Royal College of Surgeons of England and Bowel Cancer UK had no role in the study design, writing of the report, or the decision to submit the article for publication.

## Supplementary Material

zraa064_Supplementary_Data

## Notes

Presented to the Association of Surgeons in Training annual conference, Birmingham, UK, March 2020, receiving the runner-up Women in Surgery prize
