# Supplementary material for: Gender representation in editorial boards of international general surgery journals
Source: BJS Open. 2021 Apr 9;5(2):zraa064. doi: 10.1093/bjsopen/zraa064 (PMC8036998; doi:10.1093/bjsopen/zraa064)
Supplement: zraa064_Supplementary_Data [file zraa064_supplementary_data.docx]

Supporting information Tables

**Table S1.** – Included and excluded journals from list of international surgery journals, ranked according to JIF.

| JIF Rank | Journal Title | Included/excluded |
| --- | --- | --- |
| 1 | **JAMA Surgery** | **Included** |
| 2 | **Annals of Surgery** | **Included** |
| 3 | Journal of Heart and Lung Transplantation | Excluded |
| 4 | Journal of Neurology Neurosurgery and Psychiatry | Excluded |
| 5 | **American Journal of Transplantation** | **Included** |
| 6 | Endoscopy |  |
| 7 | American Journal of Surgical Pathology | Excluded |
| 8 | **British Journal of Surgery** | **Included** |
| 9 | Journal of Thoracic and Cardiovascular Surgery | Excluded |
| 10 | **Transplantation** | **Included** |
| 11 | Journal of Bone and Joint Surgery - American Volume | Excluded |
| 12 | Neurosurgery | Excluded |
| 13 | **Journal of the American College of Surgeons** | **Included** |
| 14 | Arthroscopy - The Journal of Arthroscopic and Related Surgery | Excluded |
| 15 | Bone & Joint Journal | Excluded |
| 16 | **Liver Transplantation** | **Included** |
| 17 | Clinical Orthopaedics and Related Research | Excluded |
| 18 | Journal of Neurosurgery | Excluded |
| 19 | **Diseases of the Colon & Rectum** | **Included** |
| 20 | Plastic and Reconstructive Surgery | Excluded |
| 21 | Journal of Neurointerventional Surgery | Excluded |
| 22 | Annals of Thoracic Surgery | Excluded |
| 23 | **Hepatobiliary Surgery And Nutrition** | **Included** |
| 24 | European Journal of Cardio-Thoracic Surgery | Excluded |
| 25 | **World Journal of Emergency Surgery** | **Included** |
| 26 | **Surgery for Obesity and Related Diseases** | **Included** |
| 27 | **Annals of Surgical Oncology** | **Included** |
| 28 | **European Journal of Vascular and Endovascular Surgery** | **Included** |
| 29 | Digestive Endoscopy | Excluded |
| 30 | **Obesity Surgery** | **Included** |
| 31 | **Transplant International** | **Included** |
| 32 | Jama Otolaryngology-Head & Neck Surgery | Excluded |
| 33 | Aesthetic Surgery Journal | Excluded |
| 34 | **Surgery** | **Included** |
| 35 | **EJSO** | **Included** |
| 36 | **Journal of Trauma and Acute Care Surgery** | **Included** |
| 37 | Lasers in Surgery And Medicine | Excluded |
| 38 | **Journal of Vascular Surgery** | **Included** |
| 39 | Surgical Endoscopy and Other Interventional Techniques | Excluded |
| 40 | **International Journal of Surgery** | **Included** |
| 41 | Knee Surgery Sports Traumatology Arthroscopy | Excluded |
| 42 | **Journal of Surgical Oncology** | **Included** |
| 43 | Shock | Excluded |
| 44 | JAMA Facial Plastic Surgery | Excluded |
| 45 | **HPB** | **Included** |
| 46 | Journal of Refractive Surgery | Excluded |
| 47 | **Surgical Oncology-Oxford** | **Included** |
| 48 | Journal of Neurosurgery-Spine | Excluded |
| 49 | **Colorectal Disease** | **Included** |

**Table S2.** – Included and excluded journals from list of international journals under subcategories ‘Surgery’ and ‘Transplantation’, ranked according to SJR.

| SJR Rank | Journal Title | Included/excluded |
| --- | --- | --- |
| 1 | Journal of Heart and Lung Transplantation | Excluded |
| 2 | **Annals of Surgery** | **Included** |
| 3 | **JAMA Surgery** | **Included** |
| 4 | Journal of Neurology, Neurosurgery and Psychiatry | Excluded |
| 5 | **American Journal of Transplantation** | **Included** |
| 6 | Clinical Journal of the American Society of Nephrology | Excluded |
| 7 | Current Trauma Reports | Excluded |
| 8 | American Journal of Surgical Pathology | Excluded |
| 9 | **British Journal of Surgery** | **Included** |
| 10 | **Journal of the American College of Surgeons** | **Included** |
| 11 | Bone and Joint Journal | Excluded |
| 12 | Journal of Bone and Joint Surgery - Series A | Excluded |
|  | Biology of Blood and Marrow Transplantation | Excluded |
| 13 | Acta Orthopaedica | Excluded |
| 14 | Journal of Shoulder and Elbow Surgery | Excluded |
| 15 | **Annals of Surgical Oncology** | **Included** |
| 16 | **Transplantation** | **Included** |
| 17 | Knee Surgery, Sports Traumatology, Arthroscopy | Excluded |
|  | Nephrology Dialysis Transplantation | Excluded |
| 18 | Journal of Thoracic and Cardiovascular Surgery | Excluded |
| 19 | **Journal of Trauma and Acute Care Surgery** | **Included** |
| 20 | **Journal of Vascular Surgery** | **Included** |
| 21 | Plastic and Reconstructive Surgery | Excluded |
| 22 | **Liver Transplantation** | **Included** |
| 23 | Bone Marrow Transplantation | Excluded |
| 24 | Journal of Neurosurgery | Excluded |
| 25 | Journal of NeuroInterventional Surgery | Excluded |
| 26 | Bone and Joint Research | Excluded |
| 27 | Clinical Orthopaedics and Related Research | Excluded |
| 28 | Clinical Plasma Medicine | Excluded |
| 29 | **European Journal of Vascular and Endovascular Surgery** | **Included** |
| 30 | **Surgery** | **Included** |
| 31 | Journal of Refractive Surgery | Excluded |
| 32 | **Journal of Endovascular Therapy** | **Included** |
| 33 | Aesthetic Surgery Journal | Excluded |
| 34 | **Surgery for Obesity and Related Diseases** | **Included** |
| 35 | **Surgical Endoscopy** | **Included** |
| 36 | **Obesity Surgery** | **Included** |
| 37 | **Breast** | **Included** |
| 38 | **European Journal of Surgical Oncology** | **Included** |
| 39 | Foot and Ankle International | Excluded |
| 40 | European Spine Journal | Excluded |
| 41 | European Journal of Cardio-thoracic Surgery | Excluded |
| 42 | Journal of Neurosurgery: Spine | Excluded |
| 43 | Medical Photonics | Excluded |
| 44 | **Transplant International** | **Included** |
| 45 | Neurosurgery | Excluded |
| 46 | Cytotherapy | Excluded |
| 47 | JAMA Facial Plastic Surgery | Excluded |
| 48 | JAMA Otolaryngology - Head and Neck Surgery | Excluded |
| 49 | **Journal of Gastrointestinal Surgery** | **Included** |
| 50 | Journal of Cataract and Refractive Surgery | Excluded |
| 51 | The Journal of the American Academy of Orthopaedic Surgeons | Excluded |
| 52 | Otolaryngology - Head and Neck Surgery | Excluded |
| 53 | **Journal of Surgical Oncology** | **Included** |
| 54 | Dermatologic Surgery | Excluded |
| 55 | **Surgical Oncology Clinics of North America** | **Included** |

**Table S3. -** Final top-25 lists of General Surgery journals according to JIF and SJR.

| Rank | JIF | | SJR |
| --- | --- | --- | --- |
| 1 | JAMA Surgery | Annals of Surgery | |
| 2 | Annals of Surgery | JAMA Surgery | |
| 3 | American Journal of Transplantation | American Journal of Transplantation | |
| 4 | British Journal of Surgery | British Journal of Surgery | |
| 5 | Transplantation | Journal of the American College of Surgeons | |
| 6 | Surgeons | Annals of Surgical Oncology | |
| 7 | Liver Transplantation | Transplantation | |
| 8 | Disease of Colon and Rectum | Journal of Trauma and Acute Care Surgery | |
| 9 | Hepatobiliary Surgery and Nutrition | Journal of Vascular Surgery | |
| 10 | World Journal of Emergency Surgery | Liver Transplantation | |
| 11 | Surgery for Obesity and Related Diseases | Disease of Colon and Rectum | |
| 12 | Annals of Surgical Oncology | European Journal of Vascular and Endovascular Surgery | |
| 13 | Endovascular Surgery | HPB | |
| 14 | Obesity Surgery | Surgery | |
| 15 | Transplant International | Journal of Endovascular Therapy | |
| 16 | Breast | Surgery for Obesity and Related Diseases | |
| 17 | Surgery | Surgical Endoscopy | |
| 18 | EJSO | Obesity Surgery | |
| 19 | Journal of Trauma and Acute Care Surgery | Breast | |
| 20 | Journal of Vascular Surgery | European Journal of Surgical Oncology | |
| 21 | Interventional Techniques | Transplant International | |
| 22 | International Journal of Surgery | Journal of Gastrointestinal Surgery | |
| 23 | Journal of Surgical Oncology | Journal of Surgical Oncology | |
| 24 | HPB | Colorectal disease | |
| 25 | Surgical Oncology | Surgical Oncology Clinics of North America | |

**Table S4.** - Overview of gender representation data of online journal editorial board information (anonymised).

|  | Roles | Male | Female | Unknown | Total | % Female^1^ |
| --- | --- | --- | --- | --- | --- | --- |
| Journal 1 | Editors in Chief | 1 | 0 | 0 | 1 | 0.0% |
|  | Deputy/Executive positions | 6 | 3 | 0 | 9 | 33.3% |
|  | Senior/Specialised editorial board positions | 7 | 3 | 0 | 10 | 30.0% |
|  | Wider/General editorial board positions | 66 | 22 | 0 | 88 | 25.0% |
|  | Non-academic editorial positions | - | - | - | - | - |
|  | External academic editorial positions | 13 | 5 | 0 | 18 | 27.8% |
|  | Honorary/Founding positions | - | - | - | - | - |
|  | Administrative Positions | - | - | - | - | - |
|  | **Total** | **93** | **33** | **0** | **126** | **26.2%** |
| Journal 2 | Editors in Chief | 0 | 1 | 0 | 1 | 100.0% |
|  | Deputy/Executive positions | 4 | 1 | 0 | 5 | 20.0% |
|  | Senior/Specialised editorial board positions | 46 | 8 | 2 | 59 | 18.2% |
|  | Wider/General editorial board positions | 111 | 59 | 1 | 213 | 33.3% |
|  | Non-academic editorial positions | 1 | 2 | - | 3 | - |
|  | External academic editorial positions | 35 | 2 | 1 | 38 | 5.6% |
|  | Honorary/Founding positions | 3 | 0 | 0 | 3 | 0.0% |
|  | Administrative Positions | - | - | - | - | - |
|  | **Total** | **200** | **73** | **4** | **277** | **26.7%** |
| Journal 3 | Editors in Chief | 1 | 0 | 0 | 1 | 0.0% |
|  | Deputy/Executive positions | - | - | - | - | - |
|  | Senior/Specialised editorial board positions | 46 | 3 | 0 | 49 | 6.1% |
|  | Wider/General editorial board positions | 104 | 30 | 0 | 134 | 22.4% |
|  | Non-academic editorial positions | 4 | 0 | 0 | 4 | 0.0% |
|  | External academic editorial positions | 1 | 0 | 0 | 1 | 0.0% |
|  | Honorary/Founding positions | 1 | 0 | 0 | 1 | 0.0% |
|  | Administrative Positions | - | - | - | - | - |
|  | **Total** | **157** | **33** | **0** | **190** | **17.4%** |
| Journal 4 | Editors in Chief | 0 | 1 | 0 | 1 | 100.0% |
|  | Deputy/Executive positions | 1 | 1 | 0 | 2 | 50.0% |
|  | Senior/Specialised editorial board positions | 3 | 7 | 1 | 11 | 77.8% |
|  | Wider/General editorial board positions | 27 | 20 | 8 | 55 | 51.3% |
|  | Non-academic editorial positions | 3 | 1 | 1 | 5 | 33.3% |
|  | External academic editorial positions | - | - | - | - | - |
|  | Honorary/Founding positions | - | - | - | - | - |
|  | Administrative Positions | - | - | - | - | - |
|  | **Total** | **34** | **30** | **10** | **74** | **55.6%** |
| Journal 5 | Editors in Chief | 1 | 0 | 0 | 1 | 0.0% |
|  | Deputy/Executive positions | 7 | 1 | 0 | 8 | 12.5% |
|  | Senior/Specialised editorial board positions | 0 | 1 | 0 | 1 | 100.0% |
|  | Wider/General editorial board positions | 48 | 12 | 2 | 62 | 20.7% |
|  | Non-academic editorial positions | 0 | 1 | 0 | 1 | 100.0% |
|  | External academic editorial positions | 2 | 0 | 0 | 2 | 0.0% |
|  | Honorary/Founding positions | - | - | - | - | - |
|  | Administrative Positions | - | - | - | - | - |
|  | **Total** | **58** | **15** | **2** | **75** | **21.1%** |
| Journal 6 | Editors in Chief | 1 | 0 | 0 | 1 | 0.0% |
|  | Deputy/Executive positions | 4 | 0 | 0 | 4 | 0.0% |
|  | Senior/Specialised editorial board positions | 20 | 5 | 1 | 26 | 20.8% |
|  | Wider/General editorial board positions | 4 | 0 | 2 | 6 | 0.0% |
|  | Non-academic editorial positions | - | - | - | - | - |
|  | External academic editorial positions | 22 | 4 | 10 | 36 | 25.0% |
|  | Honorary/Founding positions | - | - | - | - | - |
|  | Administrative Positions | - | - | - | - | - |
|  | **Total** | **51** | **9** | **13** | **73** | **19.1%** |
| Journal 7 | Editors in Chief | 0 | 1 | 0 | 1 | 100.0% |
|  | Deputy/Executive positions | 2 | 1 | 0 | 3 | 33.3% |
|  | Senior/Specialised editorial board positions | 6 | 1 | 0 | 7 | 14.3% |
|  | Wider/General editorial board positions | 38 | 9 | 0 | 47 | 19.1% |
|  | Non-academic editorial positions | - | - | - | - | - |
|  | External academic editorial positions | - | - | - | - | - |
|  | Honorary/Founding positions | - | - | - | - | - |
|  | Administrative Positions | 0 | 1 | 0 | 1 | 100.0% |
|  | **Total** | **46** | **13** | **0** | **59** | **22.0%** |
| Journal 8 | Editors in Chief | 1 | 0 | 0 | 1 | 0.0% |
|  | Deputy/Executive positions | 2 | 0 | 0 | 2 | 0.0% |
|  | Senior/Specialised editorial board positions | 14 | 4 | 0 | 18 | 22.2% |
|  | Wider/General editorial board positions | 95 | 34 | 1 | 130 | 26.6% |
|  | Non-academic editorial positions | - | - | - | - | - |
|  | External academic editorial positions | - | - | - | - | - |
|  | Honorary/Founding positions | 3 | 1 | 3 | 7 | 100.0% |
|  | Administrative Positions | - | - | - | - | - |
|  | **Total** | **115** | **39** | **4** | **158** | **26.0%** |
| Journal 9 | Editors in Chief | 1 | 0 | 0 | 1 | 0.0% |
|  | Deputy/Executive positions | 1 | 0 | 0 | 1 | 0.0% |
|  | Senior/Specialised editorial board positions | 8 | 3 | 0 | 11 | 27.3% |
|  | Wider/General editorial board positions | 19 | 3 | 4 | 26 | 16.7% |
|  | Non-academic editorial positions | 3 | 0 | 0 | 3 | 0.0% |
|  | External academic editorial positions | - | - | - | - | - |
|  | Honorary/Founding positions | 7 | 0 | 0 | 7 | 0.0% |
|  | Administrative Positions | 1 | 1 | 0 | 2 | 50.0% |
|  | **Total** | **40** | **7** | **4** | **51** | **16.3%** |
| Journal 10 | Editors in Chief | 1 | 0 | 0 | 1 | 0.0% |
|  | Deputy/Executive positions | 6 | 1 | 1 | 8 | 16.7% |
|  | Senior/Specialised editorial board positions | 3 | 1 | 2 | 6 | 50.0% |
|  | Wider/General editorial board positions | 65 | 5 | 0 | 70 | 7.1% |
|  | Non-academic editorial positions | - | - | - | - | - |
|  | External academic editorial positions | - | - | - | - | - |
|  | Honorary/Founding positions | - | - | - | - | - |
|  | Administrative Positions | - | - | - | - | - |
|  | **Total** | **75** | **7** | **3** | **85** | **8.9%** |
| Journal 11 | Editors in Chief | 1 | 0 | 0 | 1 | 0.0% |
|  | Deputy/Executive positions | 1 | 0 | 0 | 1 | 0.0% |
|  | Senior/Specialised editorial board positions | 30 | 3 | 19 | 52 | 21.4% |
|  | Wider/General editorial board positions | 20 | 0 | 14 | 34 | 0.0% |
|  | Non-academic editorial positions | 4 | 0 | 0 | 4 | 0.0% |
|  | External academic editorial positions | 12 | 1 | 1 | 14 | 8.3% |
|  | Honorary/Founding positions | 1 | 0 | 0 | 1 | 0.0% |
|  | Administrative Positions | - | - | - | - | - |
|  | **Total** | **69** | **4** | **34** | **107** | **10.3%** |
| Journal 12 | Editors in Chief | 1 | 1 | 0 | 2 | 50.0% |
|  | Deputy/Executive positions | 3 | 5 | 0 | 8 | 62.5% |
|  | Senior/Specialised editorial board positions | 7 | 7 | 0 | 14 | 50.0% |
|  | Wider/General editorial board positions | 24 | 6 | 0 | 30 | 20.0% |
|  | Non-academic editorial positions | 11 | 16 | 0 | 27 | 59.3% |
|  | External academic editorial positions | 2 | 19 | 0 | 21 | 90.5% |
|  | Honorary/Founding positions | 7 | 1 | 0 | 8 | 12.5% |
|  | Administrative Positions | 2 | 4 | 0 | 6 | 66.7% |
|  | **Total** | **57** | **59** | **0** | **116** | **50.9%** |
| Journal 13 | Editors in Chief | 2 | 0 | 0 | 2 | 0.0% |
|  | Deputy/Executive positions | 0 | 1 | 0 | 1 | 100.0% |
|  | Senior/Specialised editorial board positions | - | - | - | - | - |
|  | Wider/General editorial board positions | 77 | 3 | 2 | 82 | 3.8% |
|  | Non-academic editorial positions | - | - | - | - | - |
|  | External academic editorial positions | - | - | - | - | - |
|  | Honorary/Founding positions | 6 | 0 | 0 | 6 | 0.0% |
|  | Administrative Positions | - | - | - | - | - |
|  | **Total** | **85** | **4** | **2** | **91** | **4.6%** |
| Journal 14 | Editors in Chief | 2 | 0 | 0 | 2 | 0.0% |
|  | Deputy/Executive positions | 0 | 1 | 0 | 1 | 100.0% |
|  | Senior/Specialised editorial board positions | 2 | 2 | 0 | 4 | 50.0% |
|  | Wider/General editorial board positions | 84 | 6 | 0 | 90 | 6.7% |
|  | Non-academic editorial positions | - | - | - | - | - |
|  | External academic editorial positions | - | - | - | - | - |
|  | Honorary/Founding positions | 4 | 0 | 0 | 4 | 0.0% |
|  | Administrative Positions | - | - | - | - | - |
|  | **Total** | **92** | **9** | **0** | **101** | **8.9%** |
| Journal 15 | Editors in Chief | 1 | 0 | 0 | 1 | 0.0% |
|  | Deputy/Executive positions | 1 | 0 | 0 | 1 | 0.0% |
|  | Senior/Specialised editorial board positions | 22 | 9 | 0 | 31 | 29.0% |
|  | Wider/General editorial board positions | 99 | 19 | 2 | 120 | 16.4% |
|  | Non-academic editorial positions | - | - | - | - | - |
|  | External academic editorial positions | - | - | - | - | - |
|  | Honorary/Founding positions | 2 | 0 | 0 | 2 | 0.0% |
|  | Administrative Positions | 0 | 1 | 0 | 1 | 100.0% |
|  | **Total** | **125** | **29** | **2** | **156** | **19.1%** |
| Journal 16 | Editors in Chief | 1 | 0 | 0 | 1 | 0.0% |
|  | Deputy/Executive positions | 12 | 3 | 0 | 15 | 20.0% |
|  | Senior/Specialised editorial board positions | 2 | 3 | 0 | 5 | 60.0% |
|  | Wider/General editorial board positions | 63 | 21 | 0 | 84 | 25.0% |
|  | Non-academic editorial positions | 5 | 1 | 0 | 6 | 16.7% |
|  | External academic editorial positions | - | - | - | - | - |
|  | Honorary/Founding positions | 4 | 1 | 0 | 5 | 20.0% |
|  | Administrative Positions | - | - | - | - | - |
|  | **Total** | **87** | **29** | **0** | **116** | **25.0%** |
| Journal 17 | Editors in Chief | 1 | 0 | 0 | 1 | 0.0% |
|  | Deputy/Executive positions | 0 | 1 | 0 | 1 | 100.0% |
|  | Senior/Specialised editorial board positions | 3 | 2 | 0 | 5 | 40.0% |
|  | Wider/General editorial board positions | 116 | 19 | 1 | 135 | 14.1% |
|  | Non-academic editorial positions | 3 | 1 | 0 | 4 | 25.0% |
|  | External academic editorial positions | - | - | - | - | - |
|  | Honorary/Founding positions | 5 | 0 | 0 | 5 | 0.0% |
|  | Administrative Positions | - | - | - | - | - |
|  | **Total** | **128** | **23** | **1** | **152** | **15.2%** |
| Journal 18 | Editors in Chief | 2 | 0 | 0 | 2 | 0.0% |
|  | Deputy/Executive positions | - | - | - | - | - |
|  | Senior/Specialised editorial board positions | 7 | 0 | 0 | 7 | 0.0% |
|  | Wider/General editorial board positions | 50 | 6 | 0 | 56 | 10.7% |
|  | Non-academic editorial positions | - | - | - | - | - |
|  | External academic editorial positions | - | - | - | - | - |
|  | Honorary/Founding positions | 11 | 0 | 0 | 11 | 0.0% |
|  | Administrative Positions | - | - | - | - | - |
|  | **Total** | **70** | **6** | **0** | **76** | **7.9%** |
| Journal 19 | Editors in Chief | 1 | 0 | 0 | 1 | 0.0% |
|  | Deputy/Executive positions | 1 | 4 | 0 | 5 | 80.0% |
|  | Senior/Specialised editorial board positions | 13 | 8 | 0 | 21 | 38.1% |
|  | Wider/General editorial board positions | 76 | 10 | 4 | 90 | 12.2% |
|  | Non-academic editorial positions | - | - | - | - | - |
|  | External academic editorial positions | - | - | - | - | - |
|  | Honorary/Founding positions | 8 | 0 | 0 | 8 | 0.0% |
|  | Administrative Positions | 0 | 0 | 0 | - | - |
|  | **Total** | **99** | **22** | **4** | **125** | **18.8%** |
| Journal 20 | Editors in Chief | 1 | 0 | 0 | 1 | 0.0% |
|  | Deputy/Executive positions | - | - | - | - | - |
|  | Senior/Specialised editorial board positions | 31 | 5 | 0 | 36 | 13.9% |
|  | Wider/General editorial board positions | 75 | 20 | 2 | 97 | 21.5% |
|  | Non-academic editorial positions | 1 | 0 | 0 | 1 | 0.0% |
|  | External academic editorial positions | - | - | - | - | - |
|  | Honorary/Founding positions | 13 | 1 | 0 | 14 | 7.1% |
|  | Administrative Positions | - | - | - | - | - |
|  | **Total** | **121** | **26** | **2** | **149** | **17.9%** |
| Journal 21 | Editors in Chief | 2 | 0 | 0 | 2 | 0.0% |
|  | Deputy/Executive positions | 0 | 1 | 0 | 1 | 100.0% |
|  | Senior/Specialised editorial board positions | 2 | 1 | 0 | 3 | 33.3% |
|  | Wider/General editorial board positions | 84 | 11 | 4 | 99 | 12.1% |
|  | Non-academic editorial positions | - | - | - | - | - |
|  | External academic editorial positions | - | - | - | - | - |
|  | Honorary/Founding positions | 1 | 0 | 0 | 1 | 0.0% |
|  | Administrative Positions | - | - | - | - | - |
|  | **Total** | **89** | **13** | **4** | **106** | **13.3%** |
| Journal 22 | Editors in Chief | 2 | 0 | 0 | 2 | 0.0% |
|  | Deputy/Executive positions | 1 | 0 | 0 | 1 | 0.0% |
|  | Senior/Specialised editorial board positions | 26 | 6 | 0 | 32 | 18.8% |
|  | Wider/General editorial board positions | 52 | 17 | 0 | 69 | 24.6% |
|  | Non-academic editorial positions | 2 | 1 | 0 | 3 | 33.3% |
|  | External academic editorial positions | 31 | 6 | 0 | 37 | 16.2% |
|  | Honorary/Founding positions | - | - | - | - | - |
|  | Administrative Positions | - | - | - | - | - |
|  | **Total** | **114** | **30** | **0** | **144** | **20.8%** |
| Journal 23 | Editors in Chief | 2 | 0 | 0 | 2 | 0.0% |
|  | Deputy/Executive positions | - | - | - | - | - |
|  | Senior/Specialised editorial board positions | - | - | - | - | - |
|  | Wider/General editorial board positions | 7 | 2 | 3 | 12 | 33.3% |
|  | Non-academic editorial positions | - | - | - | - | - |
|  | External academic editorial positions | - | - | - | - | - |
|  | Honorary/Founding positions | 4 | 0 | 0 | 4 | 0.0% |
|  | Administrative Positions | - | - | - | - | - |
|  | **Total** | **13** | **2** | **3** | **18** | **16.7%** |
| Journal 24 | Editors in Chief | 1 | 0 | 0 | 1 | 0.0% |
|  | Deputy/Executive positions | 1 | 0 | 0 | 1 | 0.0% |
|  | Senior/Specialised editorial board positions | - | - | - | - | - |
|  | Wider/General editorial board positions | 31 | 4 | 3 | 38 | 12.5% |
|  | Non-academic editorial positions | - | - | - | - | - |
|  | External academic editorial positions | - | - | - | - | - |
|  | Honorary/Founding positions | 4 | 0 | 3 | 7 | 0.0% |
|  | Administrative Positions | 0 | 1 | 0 | 1 | 100.0% |
|  | **Total** | **37** | **5** | **6** | **48** | **13.9%** |
| Journal 25 | Editors in Chief | 2 | 0 | 0 | 2 | 0.0% |
|  | Deputy/Executive positions | - | - | - | - | - |
|  | Senior/Specialised editorial board positions | 21 | 3 | 0 | 24 | 12.5% |
|  | Wider/General editorial board positions | 33 | 3 | 9 | 45 | 11.1% |
|  | Non-academic editorial positions | 0 | 1 | 0 | 1 | 100.0% |
|  | External academic editorial positions | - | - | - | - | - |
|  | Honorary/Founding positions | 1 | 0 | 0 | 1 | 0.0% |
|  | Administrative Positions | 1 | 1 | 0 | 2 | 50.0% |
|  | **Total** | **58** | **8** | **9** | **75** | **14.0%** |
| Journal 26 | Editors in Chief | 1 | 0 | 0 | 1 | 0.0% |
|  | Deputy/Executive positions | 8 | 5 | 2 | 15 | 45.5% |
|  | Senior/Specialised editorial board positions | 2 | 2 | 1 | 5 | 66.7% |
|  | Wider/General editorial board positions | 61 | 23 | 8 | 92 | 30.3% |
|  | Non-academic editorial positions | 0 | 1 | 0 | 1 | 100.0% |
|  | External academic editorial positions | 8 | 3 | 0 | 11 | 27.3% |
|  | Honorary/Founding positions | - | - | - | - | - |
|  | Administrative Positions | - | - | - | - | - |
|  | **Total** | **80** | **34** | **11** | **125** | **33.0%** |
| Journal 27 | Editors in Chief | 2 | 0 | 0 | 2 | 0.0% |
|  | Deputy/Executive positions | 2 | 0 | 0 | 2 | 0.0% |
|  | Senior/Specialised editorial board positions | 6 | 0 | 0 | 6 | 0.0% |
|  | Wider/General editorial board positions | 36 | 5 | 5 | 46 | 13.9% |
|  | Non-academic editorial positions | - | - | - | - | - |
|  | External academic editorial positions | 6 | 1 | 3 | 10 | 25.0% |
|  | Honorary/Founding positions | 2 | 0 | 0 | 2 | 0.0% |
|  | Administrative Positions | - | - | - | - | - |
|  | **Total** | **54** | **6** | **8** | **68** | **11.5%** |

^1^ Percentage of female staff members, with ‘unknowns’ excluded from the denominator.

**Table S5.** - Gender representation data as reported by journal editorial board via email correspondence.

|  | *Roles* | *Male* | *Female* | *Total* | *% Female* |
| --- | --- | --- | --- | --- | --- |
|  |  |  |  |  |  |
| Journal 1 | Editors in Chief | 1 | 0 | 1 | 0.0% |
|  | Deputy/Executive positions | 5 | 3 | 8 | 37.5% |
|  | Senior/Specialised editorial board positions | 7 | 4 | 11 | 36.4% |
|  | Wider/General editorial board positions | 73 | 24 | 97 | 24.7% |
|  | Non-academic editorial positions | - | - | - | - |
|  | External academic editorial positions | - | - | - | - |
|  | Honorary/Founding positions | 1 | 0 | 1 | 0.0% |
|  | Administrative Positions | 0 | 3 | 3 | 100.0% |
|  | **Total** | **87** | **34** | **121** | **28.1%** |
| Journal 3 | Editors in Chief | 1 | 0 | 1 | 0.0% |
|  | Deputy/Executive positions | - | - | - | - |
|  | Senior/Specialised editorial board positions | 4 | 1 | 5 | 20.0% |
|  | Wider/General editorial board positions | 102 | 33 | 135 | 24.4% |
|  | Non-academic editorial positions | 0 | 1 | 1 | 100.0% |
|  | External academic editorial positions | - | - | 0 | - |
|  | Honorary/Founding positions | - | - | 0 | - |
|  | Administrative Positions | - | 1 | 1 | 100.0% |
|  | **Total** | **107** | **36** | **143** | **25.2%** |
| Journal 6 | Editors in Chief | 1 | 0 | 1 | 0.0% |
|  | Deputy/Executive positions | 18 | 8 | 26 | 30.8% |
|  | Senior/Specialised editorial board positions | 3 | 1 | 4 | 25.0% |
|  | Wider/General editorial board positions | 66 | 37 | 103 | 35.9% |
|  | Non-academic editorial positions | 0 | 1 | 1 | 100.0% |
|  | External academic editorial positions | - | - | - | - |
|  | Honorary/Founding positions | - | - | - | - |
|  | Administrative Positions | 1 | 1 | 2 | 50.0% |
|  | **Total** | **89** | **48** | **137** | **35.0%** |
| Journal 7 | Editors in Chief | 0 | 1 | 1 | 100.0% |
|  | Deputy/Executive positions | 2 | 0 | 2 | 0.0% |
|  | Senior/Specialised editorial board positions | 7 | 0 | 7 | 0.0% |
|  | Wider/General editorial board positions | 39 | 10 | 49 | 20.4% |
|  | Non-academic editorial positions | 0 | 1 | 1 | 100.0% |
|  | External academic editorial positions | - | - | - | - |
|  | Honorary/Founding positions | 5 | 0 | 5 | 0.0% |
|  | Administrative Positions | 0 | 1 | 1 | 100.0% |
|  | **Total** | **53** | **13** | **66** | **19.7%** |
| Journal 13 | Editors in Chief | 2 | 0 | 2 | 0.0% |
|  | Deputy/Executive positions | 0 | 1 | 1 | 100.0% |
|  | Senior/Specialised editorial board positions | - | - | - | - |
|  | Wider/General editorial board positions | 85 | 3 | 88 | 3.4% |
|  | Non-academic editorial positions | - | - | - | - |
|  | External academic editorial positions | - | - | - | - |
|  | Honorary/Founding positions | 13 | 0 | 13 | 0.0% |
|  | Administrative Positions | - | - | - | - |
|  | **Total** | **100** | **4** | **104** | **3.8%** |
| Journal 20 | Editors in Chief | 1 | 0 | 1 | 0.0% |
|  | Deputy/Executive positions | 0 | 1 | 1 | 100.0% |
|  | Senior/Specialised editorial board positions | 38 | 4 | 42 | 9.5% |
|  | Wider/General editorial board positions | 98 | 17 | 115 | 14.8% |
|  | Non-academic editorial positions | - | - | - | - |
|  | External academic editorial positions | - | - | - | - |
|  | Honorary/Founding positions | 3 | 0 | 3 | 0.0% |
|  | Administrative Positions | 1 | 1 | 2 | 50.0% |
|  | **Total** | **141** | **23** | **164** | **14.0%** |
| Journal 26 | Editors in Chief | 1 | 0 | 1 | 0.0% |
|  | Deputy/Executive positions | 10 | 5 | 15 | 33.3% |
|  | Senior/Specialised editorial board positions | 1 | 4 | 5 | 80.0% |
|  | Wider/General editorial board positions | 77 | 29 | 106 | 27.4% |
|  | Non-academic editorial positions | 2 | 2 | 4 | 50.0% |
|  | External academic editorial positions | - | - | - | - |
|  | Honorary/Founding positions | 10 | 1 | 11 | 9.1% |
|  | Administrative Positions | 1 | 1 | 2 | 50.0% |
|  | **Total** | **102** | **42** | **144** | **29.2%** |
